# Divergent primary moult—A rare moult sequence among Western Palaearctic passerines

**DOI:** 10.1371/journal.pone.0187282

**Published:** 2017-10-31

**Authors:** Yosef Kiat

**Affiliations:** Department of Ecology, Evolution and Behavior, Alexander Silberman Institute of Life Sciences, Edmond J. Safra Campus, The Hebrew University of Jerusalem, Jerusalem, Israel; Universidad de Granada, SPAIN

## Abstract

Wing morphology strongly affects flight performance which may consequently decline during feather moult due to the creation of feather gaps in the wing. Hence, the size and shape of moult-related wing gap may directly affect flight capacity. Here I examined the rare divergent primary moult sequence compared to the more common descendant moult sequence. In the divergent moult, the focus of primary moult is shifted from P_1_ (primary feather numbered descendantly) to another primary between P_2_ and P_5_, and then primaries are moulted in two concurrent waves, one descendant and the other ascendant. The result of this rare moult sequence is the splitting of the wing gap to two smaller gaps. Using a large moult database including 6,763 individuals of 32 Western Palaearctic passerine species, I found evidence of divergent moult only among 27 individuals of 12 species. I examined the speed of wing-feather moult for each individual that moulted divergently compared to a control group of individuals at the same moult stage which moulted following the common descending sequence. The results indicate that the sequence of primary moult and moult speed are correlated. Individuals which moulted divergently moulted their primaries with higher moult speed than descendant moulters. The applicability of this study is weakened by the dearth of moult data, thus making it difficult to draw conclusions for a large range of species. Ornithologists and bird ringers are therefore encouraged to collect more basic moult data during their field study.

## Introduction

The renewal of flight and body feathers is necessary to ensure future survival because old feathers become abraded and worn due to behavioural activities, exposure to sunshine and from other environmental factors [1]. All adult passerines moult their entire plumage at least once per year. All juvenile passerines also moult at least part of their plumage during their first year of life [1–3].

Fully grown feathers are dead structures consisting mainly of avian keratin. Keratin is one of the most durable biological materials, with great strength, flexibility and resistance to hydrolytic protein-digesting enzymes and bacteria [1]. However, unlike other keratin structures, such as hair and claws, feathers cannot be renewed continuously from their base and are hence replaced only following the shedding of old feathers. This shedding occurs before new replacement feathers are fully developed, and this time lag between feather shedding and the full growth of the new feather creates a feather gap. Because several adjacent feathers may be shed during a short time interval, feather gaps of various widths and lengths are created during the moult process [1,2,4]. The size of the feather gap is determined by the number of feathers that have been shed simultaneously or within a short time interval and by the feather growth rate, with the former factor being more important than the latter [5]. Moult-related feather gaps may substantially hamper flight performance and increase flight metabolism over a long period [6–10]. The aerodynamic cost of wing area reduction due to feather moult shapes the evolution of annual routine processes by dictating a slower moult speed, characterized mainly by low number of feathers that are shed simultaneously (resulting in small wing gaps) for species that regularly fly long distances and consequently, these species may be affected more substantially by large wing gaps compared with short distance flyers [11].

In addition to the moult speed, the sequence of feather moulting may also affect the size and shape of moult-related feather gaps, which in turn may also affect flight metabolism and performance during the moult period. Among Western Palaearctic passerines, the moult sequence of the primaries is generally strictly descendant starting with renewal of innermost primary (P_1_), and moving outwards [1,2]. There are only a few deviations from this moult strategy. For example, Spotted Flycatchers, *Muscicapa striata*, moult primaries in ascending sequence starting with renewal of outermost primary (P_9_) [12–14]. In addition, there are some long-distance migrants which moult some of their primaries twice a year; in this strategy the twice moulted primaries may moult in an ascending, eccentric or descending sequence [1]. Among non-passerine species, other sequences are also used for primary moult, for example, descending moult from more than one centre (e.g., Northern Gannet, *Morus bassanus*, and Common Kingfisher, *Alcedo atthis*), simultaneous moult of all primaries (e.g., Little Grebe, *Tachybaptus ruficollis*, and Mute Swan, *Cygnus olor*) and divergent sequence moult—descendant and ascendant moult from one centre (e.g., Peregrine Falcon, *Falco peregrinus*, and Common Murre, *Uria aalge*) [2,15,16].

Some passerines, which normally utilize the regular descendant sequence, may occasionally moult their primaries divergently starting with P_2-5_ instead of P_1_ [1,17]. A variable amount of Savi's Warbler, *Locustella luscinioides* (< 50.0%), and Brown Shrike, *Lanius cristatus* (9.5%), regularly show a divergent sequence, while the majority exhibit the normal descendant sequence [18–21]. The factors underlying motivation and adaptation of each primary moult sequence, descendant, ascendant or divergent, are usually overlooked. Here, using a large moult database, I examined the rare occurrence of the divergent primary moult sequence among passerines. I hypothesized that in cases of divergent sequence moult, the aerodynamic cost of wing moult is lower as a result of splitting the moult-related feather gap (two small gaps instead of one larger gap). For this reason, I predicted that the divergent sequence is correlated with higher wing-feather moult speed than the commonly used descendant sequence. The splitting of the moult-related feather gap allows birds to moult their primaries at a high speed with a reduction in the aerodynamic costs associated with moult.

## Methods

I measured active primary moult in a total of 32 Western Palaearctic passerine species. Both post-juvenile moult (only in cases where this moult involved renewal of primaries) and adult post-breeding moult were recorded. From 2006–2016, live birds were caught and sampled using mist-nets in different sites across Israel, mostly in the Beit-Shean Valley (32°29' N, 35°31' E), Judean Desert (31°32' N, 35°23' E), Mt. Hermon (33°19' N, 35°46' E), Soreq Valley (31°46' N, 34°55' E) and Jerusalem Bird Observatory (31°46' N, 35°12' E). European Starling, *Sturnus vulgaris*, moult data were collected at the Ottenby Bird Observatory, Southern Öland, Sweden (56°11' N, 16°23' E) from 2000–2013. Additional data were obtained from bird specimens stored at the Steinhardt National Collections of Natural History at the Zoological Museum of Tel-Aviv University, Israel and the Natural History Museum in Tring, UK. The field work for this study was performed under a regular ringing permit which was provided by Israel Nature and Parks Authority (NPA).

I used the primary score (PS) method to describe the moult stage of each primary feather (P_1-9_; numbered descendantly, from inside to outside, towards the wing-tip) on a scale of 0 to 5 [2] as follows: 0—a remaining old feather, 1—a missing old feather or a new feather that is found completely within its pin, 2—a new feather just emerging from its sheath up to the length of a one third of a fully grown feather, 3—a new feather with a length between one and two thirds of a fully grown feather, 4—a new feather that is more than two thirds the length of a fully grown feather and with remains of waxy sheath at its base, and 5—a new, fully developed feather with no traces of remaining waxy sheath at its base. By this method, each individual could be characterized as moulting using either the regular descendant sequence or the divergent sequence.

### Moult speed quantification

To estimate the moult speed of each individual, I estimated the size of moult-related wing gap based on the residual raggedness value (RRV) method [22], a method which based on the commonly used RRV method [11,23]. This method estimates the relative gap size in the primary feathers that is created by moult and is also strongly and negatively correlated with moult speed and duration [24]. This value is the inverse of the PS for each of the wing’s nine primary feathers (P_1-9_), such that when PS = 1, gap size = 4, and when PS = 2, gap size = 3, etc., but for PS = 0 and PS = 5, gap size = 0 because during these two moult stages there is no gap as the old and new feathers are fully grown. The total value for each individual is calculated by summing the values from each of the primary feathers. This estimate is independent of bird size and morphology, controls for the stage of wing feather moult and allows for reliable cross-species comparison [22].

### Statistical analysis

The moult speed (moult-related wing gap) of each individual that moulted primaries divergently was compared with Confidence Interval (CI; 95%) for moult speed mean of a control group that utilized the more common sequence of descendant moult. In order to include only individuals at the same moult stage, each control group included only individuals with same sum of PSs ± 5. For example, an individual undertaking divergent moult with PSs of 2-4-5-5-3-1-0-0-0 (P_1_ to P_9_; sum PSs = 20) was compared to a control group that included individuals of the same species and age undertaking a descendant primary moult with sum PSs of 15–25. The minimum number of individuals per control group was five. Analyses were performed using SPSS (version 22).

## Results

The moult database included a total of 6,763 individuals from 32 Western Palaearctic passerine species: 2,218 individuals from 32 species were sampled during the post-breeding moult (adults) and 4,545 individuals from 25 species were sampled during the post-juvenile moult (Table 1). These moult data indicate that only 27 individuals moulted their primaries divergently (< 0.4%). These individuals included three adults of three species (on average, 0.18% per species) and 24 juveniles of nine species (on average, 1.96% per species) (Table 2).

**Table 1:** 

Species	Sample size				
	Adult		Juvenile		
	N	Evidence of divergent sequence	N	Evidence of divergent sequence	Total
*Eremophila alpestris*	6	-	4	-	10
*Ammomanes deserti*	66	-	102	-	168
*Ptyonoprogne fuligula*	36	-	-	-	36
*Hirundo rustica*	327	-	801	-	1128
*Turdus merula*	62	-	6	2 (33.3%)	68
*Pycnonotus xanthopygos*	36	-	299	-	335
*Cisticola juncidis*	6	-	20	-	26
*Prinia gracilis*	27	-	201	5 (2.5%)	228
*Cettia cetti*	90	-	7	-	97
*Acrocephalus stentoreus*	80	-	141	3 (2.1%)	221
*Sylvia curruca*	32	-	-	-	32
*Sylvia melanocephala*	19	-	8	1 (12.5%)	27
*Lanius excubitor*	21	-	15	-	36
*Lanius senator*	25	-	9	-	34
*Lanius collurio*	37	-	8	-	45
*Lanius isabellinus*	26	-	26	-	52
*Lanius nubicus*	11	-	-	-	11
*Nectarinia osea*	6	-	51	-	57
*Sitta neumayer*	22	-	-	-	22
*Sturnus vulgaris*	504	-	2259	4 (0.2%)	2763
*Passer moabiticus*	59	-	159	-	218
*Passer hispaniolensis*	7	-	136	5 (3.7%)	143
*Passer domesticus*	27	-	135	2 (1.5%)	162
*Carpodacus synoicus*	12	-	-	-	12
*Rhodospiza obsoleta*	8	-	41	-	49
*Bucanetes githagineus*	21	-	26	1 (3.8%)	47
*Carduelis cannabina*	143	1 (0.7%)	22	-	165
*Carduelis carduelis*	74	-	32	1 (3.1%)	106
*Carduelis chloris*	21	1 (4.8%)	32	-	53
*Serinus syriacus*	306	1 (0.3%)	5	-	311
*Emberiza striolata*	46	-	-	-	46
*Emberiza cia*	55	-	-	-	55
*AVERAGE*	2218	0.18%	4545	1.96%	6763

The sample size of species included in the database used in this study and the evidence for the divergent primary moult sequence.

**Table 2:** 

Ring Number	Age	Moult Score									Size of moult-related wing gap	Size of moult-related wing gap for control group (mean ± SD)	95% Confidence Interval for size of moult-related wing gap mean (control group)	
		P_1_	P_2_	P_3_	P_4_	P_5_	P_6_	P_7_	P_8_	P_9_			Lower bound	Upper bound
Blackbird (*Turdus merula*)														
C-30726	Juv	2	3	3	2	0	0	0	0	0	10	-	-	-
C-56791	Juv	3	4	1	0	0	0	0	0	0	7	-	-	-
Graceful Prinia (*Prinia gracilis*)														
2TE-0840	Juv	0	0	3	4	4	3	1	0	0	10	5.66 ± 1.79 (n = 65)	5.22	6.10
RC-3128	Juv	2	3	1	0	0	0	0	0	0	9	5.94 ± 2.00 (n = 48)	5.36	6.52
RC-3129	Juv	1	2	1	0	0	0	0	0	0	11	6.03 ± 2.01 (n = 36)	5.35	6.71
RC-3169	Juv	0	4	5	5	5	3	2	1	0	10	6.02 ± 1.55 (n = 47)	5.57	6.48
RC-3185	Juv	1	3	1	0	0	0	0	0	0	10	5.90 ± 1.91 (n = 41)	5.30	6.51
Clamorous Reed-Warbler (*Acrocephalus stentoreus*)														
AB-51413	Juv	2	2	5	5	4	2	1	0	0	14	5.39 ± 2.32 (n = 36)	4.60	6.17
AB-51415	Juv	2	2	5	5	4	2	0	0	0	10	5.71 ± 2.46 (n = 34)	4.85	6.56
AB-51442	Juv	3	3	5	5	5	3	1	0	0	10	5.55 ± 2.50 (n = 31)	4.63	6.47
Sardinian Warbler (*Sylvia melanocephala*)														
X-370602	Juv	0	0	3	4	5	4	2	0	0	7	-	-	-
European Starling (*Sturnus vulgaris*)														
4483071	Juv	2	3	1	0	0	0	0	0	0	9	7.26 ± 1.95 (n = 479)	7.08	7.43
4484339	Juv	1	5	5	5	5	1	0	0	0	8	4.32 ± 1.57 (n = 1538)	4.24	4.40
4505604	Juv	1	5	5	5	4	3	0	0	0	7	4.33 ± 1.66 (n = 1663)	4.25	4.41
4505606	Juv	3	5	5	5	5	4	1	0	0	7	4.28 ± 1.40 (n = 1281)	4.20	4.36
Spanish Sparrow (*Passer hispaniolensis*)														
BB-36233	Juv	2	5	4	3	1	0	0	0	0	10	5.42 ± 1.88 (n = 67)	4.96	5.88
AC-10075	Juv	2	4	1	0	0	0	0	0	0	8	5.35 ± 1.99 (n = 51)	4.79	5.91
AC-10085	Juv	4	5	4	3	1	0	0	0	0	8	5.50 ± 1.98 (n = 60)	4.99	6.01
AC-11004	Juv	4	5	5	4	3	1	0	0	0	8	5.50 ± 1.69 (n = 34)	4.91	6.09
AC-11008	Juv	4	5	5	4	2	0	0	0	0	5	5.43 ± 1.73 (n = 46)	4.92	5.95
House Sparrow (Passer domesticus)														
AB-80880	Juv	2	5	1	0	0	0	0	0	0	7	4.63 ± 2.41 (n = 52)	3.96	5.31
AB-80885	Juv	2	5	1	0	0	0	0	0	0	7	4.63 ± 2.41 (n = 52)	3.96	5.31
Trumpeter Finch (*Bucanetes githagineus*)														
BG-49614	Juv	0	0	4	5	3	0	0	0	0	3	-	-	-
Syrian Serin (*Serinus syriacus*)														
Y-212876	Ad	3	4	2	1	0	0	0	0	0	10	7.48 ± 2.42 (n = 128)	7.05	7.90
European Greenfinch (*Carduelis chloris*)														
V-23094	Ad	2	4	4	2	0	0	0	0	0	8	4.57 ± 2.51 (n = 7)	2.25	6.89
European Goldfinch (*Carduelis carduelis*)														
V-18585	Juv	2	3	2	1	0	0	0	0	0	12	4.45 ± 2.70 (n = 11)	2.64	6.27
Common Linnet (*Carduelis cannabina*)														
X-131664	Ad	4	5	5	4	1	0	0	0	0	6	5.56 ± 2.04 (n = 18)	4.54	6.57

The primary moult score, size of the moult-related wing gap and CI (95%). The CI values are only provided for the 23 out of 27 individuals that had a relevant control group (> 5 individuals).

**Fig 1:**
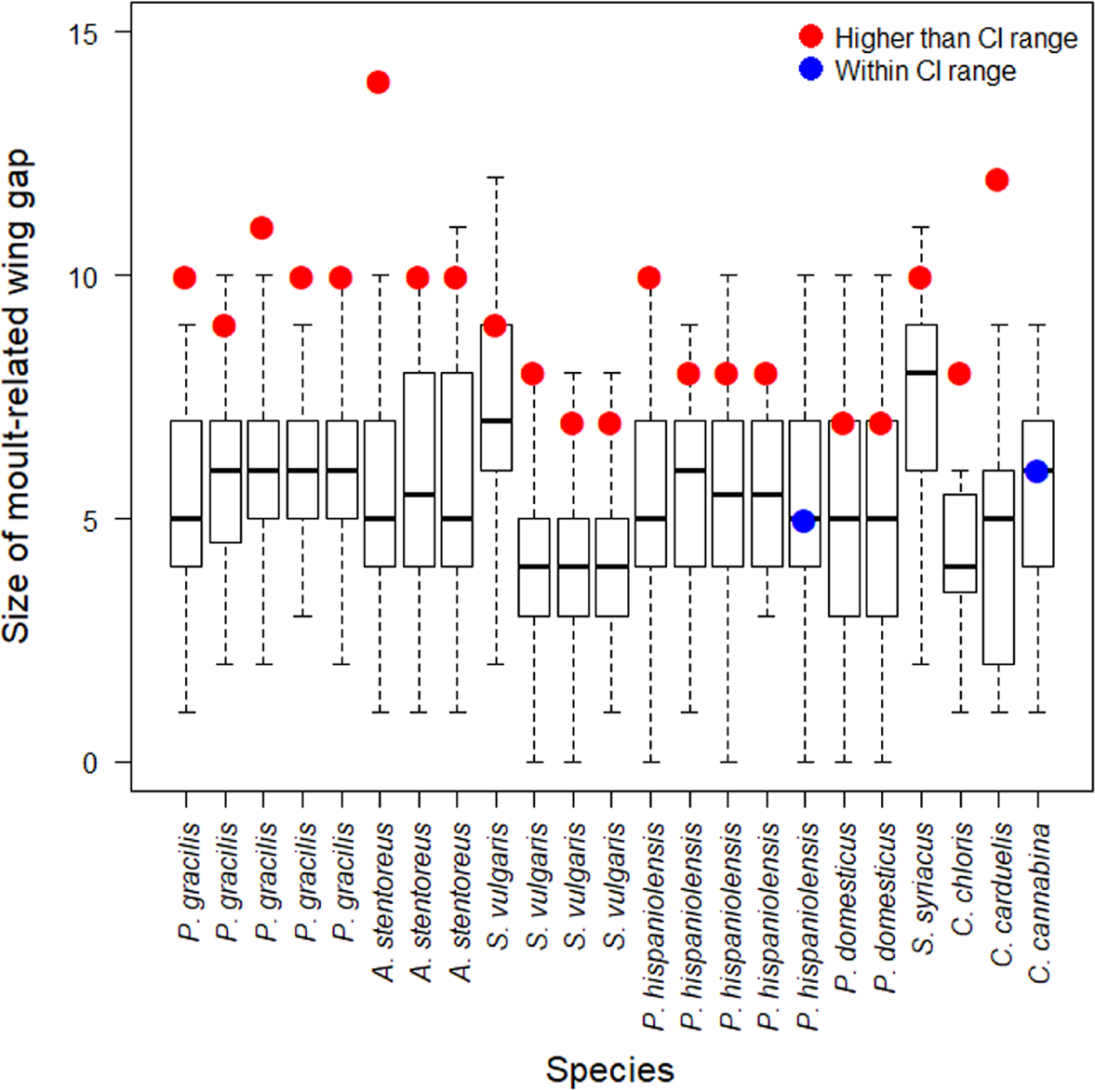
The size of moult-related wing gap (moult speed) of the 23 individuals that undertook a divergent moult as compared with relevant control groups. The red circles represent the moult speed of individuals that moulted divergently at higher speeds than CI range (95%) of individuals showing the regular descendant sequence. The boxplots display the minimum, 1^st^ and 3^rd^ quartile, median and maximum values for each control group.

From the moult database, control groups (> 5 descendant moult individuals) could only be constructed for 23 of the 27 individuals that moulted divergently. Twenty-one of the divergent sequence moulters included in analysis (n = 23) moulted their primaries at a higher speed than the more common descendant sequence moulters (on average 33.0% higher than descendant sequence moulters; Table 2 and Fig 1).

## Discussion

**Fig 2:**
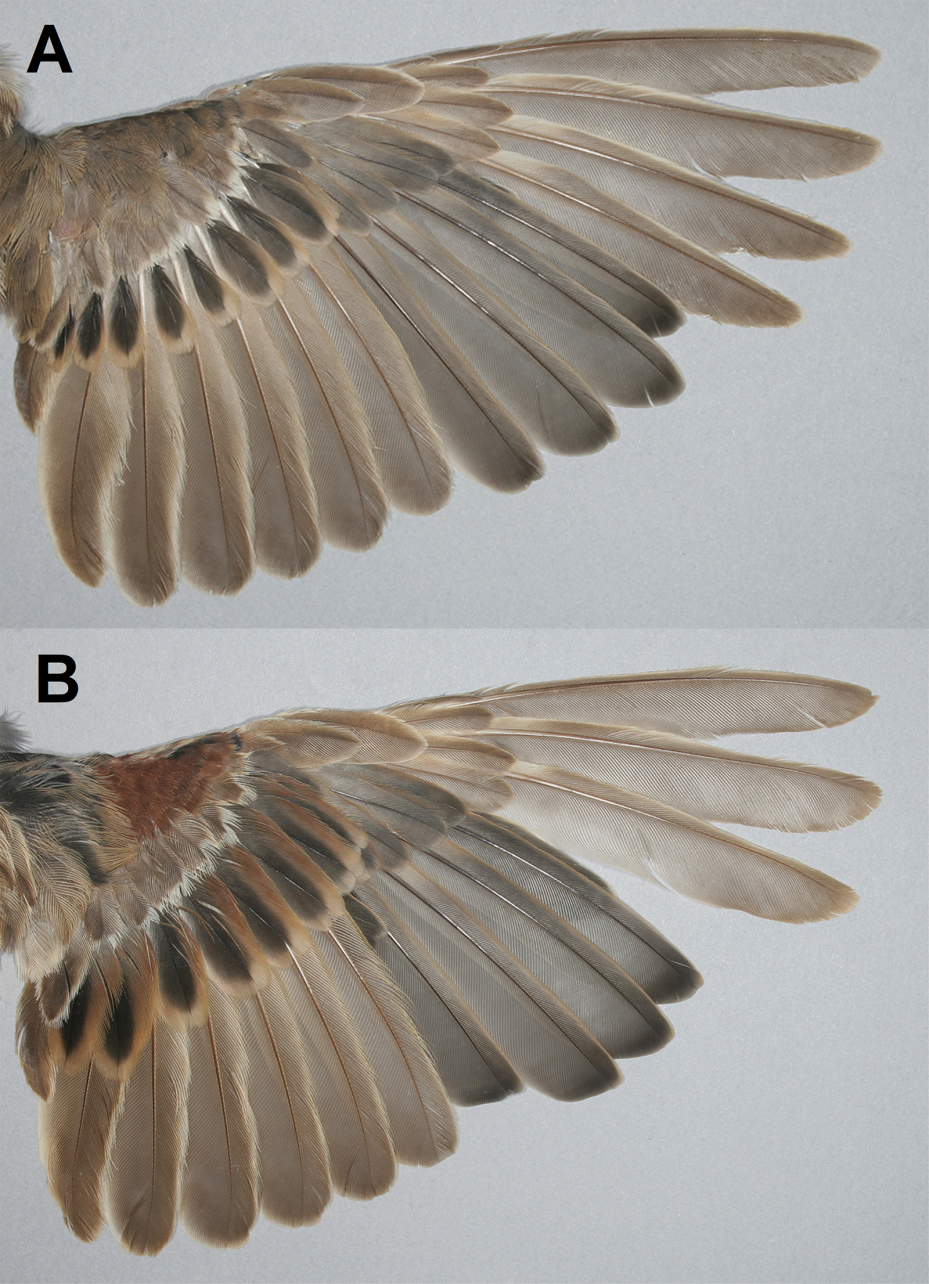
Two Spanish Sparrow, *Passer hispaniolensis*, juveniles in the post-juvenile moult: **(A)** primaries moulted in a descendant sequence and the relative size of the moult-related wing gap = 5 (primary moult score: 5-5-4-4-2-0-0-0-0) and **(B)** primaries moulted divergently and the relative size of the moult-related wing gap = 8 (primary moult score: 4-5-5-4-3-1-0-0-0; individual with ring number AC-11004 from Table 2).

The present study investigated the rarely utilized divergent primary moult sequence among Western Palaearctic passerines. With this strategy, the focus of primary moult shifted from P_1_ (descendant moult) to another primary (P_2-5_) and remaining primaries were moulted in both descending and ascending sequences (for example see Fig 2). This may reduce the effect of moult on flight by splitting the moult-related wing gap. During the descendant moult sequence (regular sequence), there is one larger wing gap during the first part of moult, which remains until the ascending secondaries moult, usually beginning during growth of the P_5_ [1]. After the onset of the secondaries' ascendant moult, there is a splitting of the wing gap into two smaller gaps. In the case of divergent primary moult, the splitting of the wing gap into two smaller gaps occurs earlier, shortly after the start of the moult of primaries. Study of the impact on flight of different moult sequences, descending or divergent, is a logical next step for future research.

The results indicate that the moult-related wing gap of most individuals which moulted divergently is larger than of individuals that moulted following a descending sequence (21 out of 23 individuals; Table 2 and Fig 1). The divergent moult tended to be slightly less rare among juveniles (1.96%) than among adults (0.18%) (Table 1). This result may be indicative of a greater benefit of moulting divergently for juveniles than adults. A previous study showed that adults moult their primaries with higher speed than juveniles (larger moult-related wing gap). These results may emphasizes the higher environmental constraints on juveniles than on adults during primary moult [23], likely due to the lower foraging success [25] and higher predation risk of juveniles. If the divergent moult allows for higher moult speed, but with lower aerodynamic or energetic costs, this could be more adaptive for juveniles than for adults. But, it is still not clear if there is a cost associated with this strategy and why it is so rare among passerines.

The costs of moult itself may limit moult speed as this process has a direct energy cost simply due to the production of new feathers [26]; the size of the feather follicle constrains the speed at which feathers can be generated [5]. In addition, there are indirect costs associated with elevated energy expenditure during flight due to reduced wing area and the heightened risk of being preyed upon because of lowered flight performance [6]. However, the magnitude of each factor’s impact on moult speed is still unclear. Perhaps, the individuals that moulted their primaries divergently represent a case in which there is an abundance of resources, thus providing the energy required to moult more feathers simultaneously. Moulting more feathers simultaneously in descendant sequence, though, is impossible because it is likely associated with high flight costs due to reduced wing area [8,9], and hence the splitting of the moult-related wing gap to two smaller gaps by divergent moult may be a suitable solution (as found in some species with numerous secondaries [2,16]). On the other hand, the fact that divergent moult is such a rare moult sequence among Western Palaearctic passerines may indicate that, in general, the main limitation on moult speed stems from the direct energy costs needed for the production of new feathers [26]. Thus, the results could indicate a correlation between flight performance and moult speed [11] likely stemming from the different habitat preferences between species and from the indirect costs of increased predation pressures as a result of reduced wing area and thus, flight ability [8,27,28].

Feathers are the unifying characteristic of all birds, yet our understanding of moult strategies and plumage lags behind that of other major life history phenomena. This study broadens our understanding of feather moult by focusing on a rare moult sequence strategy in passerines. Application of findings of this study across species is challenged mainly by availability of moult data. For many species, comprehensive information about feather moult is lacking. Documentation of basic moult data such as timing, location, sequence, intensity, completeness, and degree of individual variation for many species is still missing. Newton [3] and Bridge [29] have already been plagued by these gaps in the moult data. I join these researchers in encouraging ornithologists and bird ringers to collect more basic moult data during their field study.
